# Book Reviews

**Published:** 1824-09

**Authors:** 


### CRITICAL ANALYSIS
### OF
### ENGLISH AND FOREIGN LITERATURE
### RELATIVE TO THE VARIOUS BRANCHES OF
### Medical Science.

Quæ laudanda forent, et quæ culpanda, vicissim

Illa, prius, cretâ; mox hæc, carbone, *notamus*.—PERSIUS.

### DIVISION I.

### ENGLISH.

ART. I.—*The Principles of Forensic Medicine systematically arranged, and applied to British Practice.* By JOHN GORDON SMITH, M.D. Lecturer on Political Medicine. Second Edition, greatly enlarged.—8vo. pp. 569. Underwood, London, 1824.

[Concluded from page 148.]

WE have already adverted to some useful rules for the direction of the practitioner in his medico-legal capacity; these are more particularly detailed and applied in treating of the white oxide of arsenic, or *arsenious acid.* If we have not arrived before the death of the patient, or our efforts to effect recovery prove unavailing, our duty then is confined to determining the cause of death.

There are three circumstances to which the practitioner has to look to for proofs of poisoning by arsenious acid, and, indeed, of poisoning in general:—1. The symptoms under which the patient laboured; 2, the morbid appearances observed on dissection after death; and 3, the chemical examination of the contents of the alimentary canal, or of the matters which may have been ejected from the stomach before death. Our author gives an accurate description of the symptoms which usually characterize the action of arsenic, and the corresponding morbid alterations of structure discovered in the body on dissection: but these, he observes, however marked they may be, are not to be considered, alone, as sufficient evidence that poison was the fatal agent. Symptoms of a formidable character, and very analogous to those induced by poisons, sometimes result from diseases arising from other causes, which will occasion changes of structure, not easily distinguishable from those produced by mineral poisons.

The detection, therefore, of the poisonous article itself is the only indisputable proof of poisoning, and ought in every instance to be the grand object of our research. With this view we are to search for solid particles of the mineral in the stomach and intestines; and, if successful, submit it to the action of the usual chemical reagents. The fluid and solid contents of the alimentary canal we must also treat in the same manner, not resting satisfied till we have finally reduced the poison to the metallic state. The production, by tests, of precipitates strictly answering to the account given of each, is but a preliminary step in the detection of arsenic: from all of them, when dried and calcined with potass and charcoal, the metal itself is to be obtained; and to procure this our labours must be directed. We must pass over what relates to the different chemical reagents employed as tests of arsenic, and will conclude this part of the subject by quoting the author's directions for conducting the inspection of the body in search of morbid appearances and the presence of the poison.

The practitioner, on such important occasions, should obtain the presence and co-operation of another professional man as soon as possible; and, if he is unaccustomed to chemical operations, he should avail himself of the assistance of some more expert or experienced person.

Although no great importance is to be attached to the external appearances presented by the bodies of those who have died from the action of arsenic, we are not to overlook them, and it is our duty to note those which may seem unusual. The interior research, however, is the important consideration, and this we cannot perform too scrupulously.

"The trunk of the body is to be carefully laid open, from the top of the thorax to the cavity of the pelvis, taking every precaution to wound no part of the alimentary canal. This being done, let the whole of the intestines be removed; which is to be accomplished by careful separation from their attachments; placing one ligature securely on the upper part of the œsophagus, a second on the lower part of the intestinum rectum, and a third on the vessels that pass between the duodenum and the liver, whereby every possible precaution will be taken to guard the contents from escape. If we discover preternatural perforations in the stomach or elsewhere, ligatures, even if practicable, might be improper: we must endeavour to avoid the loss of substance through them, by attending to the position in which they are maintained during the process of dissection; and clean sponges may be applied, to prevent, as much as possible, the fluid from spilling, and, by absorbing, to preserve what portion does make its way through.

"While this is going on, a large earthen vessel, of a capacity sufficient to receive the viscera, should be prepared, perfectly clean and dry, to which the whole intestinal canal is to be transferred without delay.

"Other vessels of the same kind, though not necessarily of equal dimensions, are also to be got ready; and the alimentary canal being laid open throughout its whole extent, the fluid contents are to be placed in one vessel, and the solid in another. The intestines are then to be washed in warm distilled water, and the product of this also is to be carefully set apart. These precautionary steps being taken, we proceed to search accurately for lesions of structure, and morbid appearances; and whatever may be discovered in this way should be correctly noted. Eschars, gangrenous, inflamed, and perforated spots, Orfila recommends to be removed, with a portion of the parts around them, and placed in alcohol.

"The preliminary preparations for a chemical examination having been arranged, we now proceed to analyze the various substances obtained. In the first place, we must search for solid particles of the arsenious acid, and, if we find any, let them be tried in various ways. If the search for these be unavailing, our attention must be directed to the contents of the alimentary canal in general; and it will be a convenient rule to keep those of the stomach separate from the rest.

"M. Orfila directs that the solid part of the contents should be boiled in ten or twelve times its weight of distilled water for one hour, renewing the water as fast as it flies off in vapour. This liquor is to be cooled, and decanted from the residue before the tests be applied. But, as the degree of solubility of arsenious acid in water at the boiling point, is stated by Orfila himself to be as one part to fifteen of water,* I should think that the success of the experiment would be better insured, were the quantity of water greater than here recommended, even although the proportion of arsenic contained in the mass to be boiled should be small.

"With regard to the application of tests, the safest rule will be to divide the substance to which they are to be applied into separate portions, and the more tests we apply upon the whole, the better; provided we apply them properly, and do not dispose too liberally of a quantity of the suspected substance, insufficient to enable us to proceed all possible lengths with it. We shall often find portions of the poison unchanged. The fluid part of the substance rejected from the stomach should be filtered, and the tests applied as to any other solution. The solid matter may be partly dissolved in distilled water, filtered, and tried in the same manner; or dried and submitted to the test of heat; to which, indeed, the precipitates themselves, obtained in the other steps of the experiment, should ultimately be referred, for this is the only sure method; the object being not to produce merely precipitates of certain colours, by the application of certain articles.

"The final result of the reduction of arsenious acid to the metallic state, may be obtained by simply evaporating the fluid to dryness, and subliming the residue. This may be very properly done with a portion of it, where it is abundant enough to admit of variety in the experiments. But, in all cases, the practitioner will do wisely to apply the chemical tests, and note accurately the results of precipitation, as to rapidity of appearance, quantity, and colour.

"But we have not yet exhausted the whole of our means of detection. The judgment of the practitioner, with regard to the success of the processes already detailed, may lead him to decide that he need not

---

* *Toxicology*, vol. i. p. 103.

proceed with what remains to be explained. It will be recollected that it was enjoined to preserve the portions cut from the intestines in alcohol. If all our experiments on their contents fail, we should take these portions themselves, and, after drying them, combine them with potash and charcoal, and subject them to heat, with a view to obtain metallic arsenic by sublimation.

" The great, the safe, and indeed the inevitable, principle to be kept in the mind of the practitioner, with regard to the detection of metallic poisons, and of arsenic in particular, is this:—Any one test is but corroborative of the rest; therefore, a plurality should be employed, from the concurrent results of all which only will his opinion be warrantable, and receivable as evidence: and that the metallization, where the quantity of the poison is sufficient to enable him to go that length, must never be omitted; otherwise, his conclusions will be subject to animadversion, at least, if not to rejection. It was upon this point that the important trial at Launceston turned, and the principal evidence, though perhaps (in the estimation of scientific men) satisfactory enough, went for nothing in that of the jury." (Pp. 98—105.)

The other preparations of a metallic nature, which possess a poisonous quality, are treated in the same manner as those of arsenic. Many of them are extensively employed in medicine, and unfortunately, when imprudently administered, have sometimes been found to act as poisons, instead of antidotes: some have been employed with criminal intentions, and numerous instances of serious consequences have resulted from others being swallowed through accident or inattention. The mode of proceeding in the detection of arsenic is applicable to them. The peculiarities in the chemical and toxicological history of each individual article are discussed by the author, with the requisite minuteness and precision.

The concentrated acids, the sulphuric, nitric and muriatic, as objects of toxicology, are next considered; and the alkalies, earths, and the neutral salt, nitre, conclude the subject of mineral poisons.

The *vegetable* kingdom contains more individual poisons than all the rest together, but they are less employed, either with a criminal intention or for the purpose of self-destruction, than those of the mineral kingdom. The taste, colour, and other sensible qualities belonging to plants, throw an obstacle in the way of attempting the life of others; and the comparative difficulty of collecting them, and afterwards preparing them into poisons, prevent their being so frequently resorted to by those who are weary of existence. They are often, however, the accidental cause of death.

In investigating vegetable poisons, with the view of ascertaining the particular article concerned, the practitioner will meet with difficulties which are not presented by the mineral poisons.

By chemical processes he may be able to resolve the vegetable into its elementary principles, and yet not be able to say more than that vegetable matter is concerned. Analysis will not enable him to determine the particular plant on which he is operating, and synthetical proofs fail him entirely: the constituent principles he may obtain; but he cannot, as in the case of minerals, recombine these so as to reproduce the original plant. Here, as our author observes, organization opposes a barrier to our creative powers.

Notwithstanding, however, that in our researches we are so far deprived of the aid which chemistry affords in the other case, the investigation may be carried on with less trouble, and, in most cases, may be brought to a sufficiently satisfactory conclusion, by attending to certain circumstances connected with the nature of vegetable poisons, and their effects on the constitution. The practitioner should, therefore, be well acquainted with the habitats, the botanical characters, as well as the sensible qualities, of plants belonging to this department, both in the recent and organized state, and in the various forms of powder, tincture, extract, &c. which they are usually made to assume.

The sensible properties of vegetables (which furnish the principal means of detection) are not so readily destroyed as those of minerals: they remain longer unchanged in the alimentary canal,—a peculiarity which affords not only a better chance of recovery to the patient, but greater facility of recognizing the deleterious article in the matter vomited, or contained in the stomach and intestines. Vegetable poisons also differ from the minerals, in not acting chemically on the living solids to which they are applied; not destroying organic texture, otherwise than through the medium of the inflammation which those of an acrid nature excite; no article of this kingdom (if we except one, oxalic acid, derived from it,) being referable to the class of corrosive poisons: they are, consequently, more simple in their effects. Some of them, as the lauro-cerasus, act immediately and powerfully on the nervous system, proving speedily fatal, without leaving any discoverable trace of their action. Symptoms, in this case, claim more consideration as proofs of poisoning, than when we are dealing with articles of the mineral kingdom, where the certainty resulting from the application of chemical tests renders other modes of investigation less essential.

The individual articles belonging to the vegetable kingdom are discussed in the order of the six classes to which the mineral poisons were referred. The most important of these classes is the narcotic; and opium, being the article which most frequently forms the subject of medico-legal inquiry, holds the first rank. The means of detecting this poison resolve themselves into the observation of symptoms, examination of the ingesta, morbid appearances, and the history of the case. The post-mortem appearances do not of themselves afford any very satisfactory proof of the agency of this poison, but, in a pathological and practical point of view, they are important. Slight marks of inflammation of the stomach have been discovered in those who have been poisoned by this drug, but the most prominent phenomenon has been general congestion of blood in the internal organs. The brain, lungs, and heart are described as *loaded* with black blood. These appearances, and the corresponding symptoms during life, undoubtedly indicate the propriety of abstracting blood; and some recent cases of the successful application of this practice are referred to by the author.

The discussion of *animal* and *septic poisons*, in the relation which they bear to medical jurisprudence, concludes the Chapter on Poisoning. In this respect they are less important than those of the two preceding departments of nature, and consequently are not so fully treated of.

The Chapter on *Suffocation* includes every variety of death resulting from impeded respiration, whether induced by exposure to another aerial medium than that of the respirable atmosphere, as in the case of *noxious inhalations*, or to a denser medium which cannot be admitted by the organs of respiration, as in *drowning*, or by mechanical obstruction to the admission of the respirable air into the lungs, as in *hanging*, *strangling*, and *smothering*.

When respiration is impeded under any of these circumstances, unoxygenated blood is sent to the brain, upon which it exerts a most deleterious influence: in a short time the circulation through the lungs is arrested, and accumulation of blood takes place in the venous system, while the arterial is empty. This theory of the mode of death in suffocation is supported by the appearances observed on dissection. The lungs are found of a deep-blue colour, gorged with blood, with extravasation into the air-vessels; a similar congestion is found in the right cavities of the heart, extending to the neighbouring veins, the cavæ, the jugulars, and their ramifications, causing turgescence, and sometimes rupture, of the vessels of the brain, and lividity of the surface, particularly of the face and breast.

The notice which our author gives of the noxious gases is rather too brief, considering the frequency of death from these agents, and the judicial and professional investigation to which they give rise. Such occurrences are generally made the subject of inquiry before the coroner, and sometimes a suspicion of murder is attached to them, till medical testimony explains the real nature of the event.

Our author's observations are chiefly confined to carbonic acid. This gas is produced naturally in some places, to a sufficient amount to prove fatal; but its deleterious effects are more ordinarily witnessed where the combustion of charcoal is going on without the renewal of atmospheric air,—as in close apartments, in the cabins of ships, in lime-kilns, &c.; it is also accumulated to a dangerous extent in cellars, wells, breweries, and green-houses; it forms the *choak damp* of coal-mines; and, as the product of animal respiration, it is the cause of the deterioration of the atmosphere of confined places in which a number of people are crowded together, without an adequate supply of fresh air, as happened in the catastrophe of the Black-hole at Calcutta; and in this country a few years before that event, when the keeper of St. Martin's watch-house forced twenty-eight persons, of whom four were suffocated, into a place called the Hole, not above six feet square, and scarce five feet ten inches high.

Various opinions prevail as to the manner in which this gas proves fatal. Some maintain that it is not inspired, but causes a constriction of the glottis, and kills in a way analogous to drowning. This is true of the pure gas, which Sir H. Davy was never able in his experiments to inspire, when unmixed with common air; but, when diluted with atmospheric air, it may be breathed, and in general, when it proves fatal, it is thus diluted. Some suppose that, when inspired, it acts negatively, by excluding the oxygenated air; and this opinion is in some degree supported by the circumstance, that the subject frequently recovers as soon as he is removed into fresh air: but there are other observations which favour an opposite opinion, that it is positively deleterious,—particularly the cases related by Dr. Babington and Dr. King, in which the effects of charcoal vapours were very general and permanent, and in some of them ultimately fatal.

During the combustion of humid charcoal, besides carbonic acid, there is evolved another gaseous compound, hydrocarbonous acid, or the heavy inflammable air and hydrocarbonate of the earlier chemists. This gas is decidedly deleterious, as appears from the experiments of Sir H. Davy, who, with a boldness bordering on rashness, inspired it nearly pure. In him it produced very alarming effects: on the first inspiration, numbness of the chest, with head-ache; then dreadful oppression and insensibility; and on the third inspiration he thought he was sinking into annihilation, and had only power to drop the mouth-piece from his unclosed lips. These effects were not merely transient; very disagreeable sensations remained for several days.

Our author does not take any notice of carburetted hydrogen, the *fire-damp* of coal-mines, and which is frequently the cause of fatal accidents, by exploding when ignited, and suffocating when inspired; nor of sulphuretted hydrogen, which is generated in such abundance in sewers, privies, &c. as to prove dangerous to persons employed in emptying them.

In treating of *drowning*, our author, after enumerating the ordinary appearances observed in the bodies of those who have been drowned, and giving what is at present considered to be the most correct view of the *ratio moriendi* in this kind of death, proceeds to consider the subject in its relations to forensic medicine, or the various questions which, in cases of doubt, require medical testimony for their solution. It may, in this way, become matter of inquiry whether a body, found under circumstances leading to a suspicion of drowning, was really drowned, or placed in the water after death; whether the person was first killed, and then thrown into the water by the assassin, to conceal the crime; or whether the deceased fell into the water subsequent to death from accident, disease, or suicide; and, lastly, whether the drowning (supposing the fact to be satisfactorily established) was voluntary, accidental, or forced.

The most important of these is, whether the person was alive or dead when submersed; a point which, in the absence of moral evidence, is to be determined by a careful examination of the body, with the view of discovering the usual marks of death from drowning, or such indications as may prove it to have been caused in some other way.

This subject has given rise to much discussion in courts of justice, and great discrepancy of opinion among professional witnesses. The difficulty of coming to a right conclusion is chiefly owing to the uncertainty of the signs of drowning; a difficulty acknowledged by all writers on the subject, and which has led some to think that the decision of such questions is not determinable by medical testimony. We were, therefore, not prepared for the observation of our author, "that great difficulty need not occur in declaring whether a person has been submersed while alive, or thrown into the water after death;" a conclusion scarcely warranted by the preceding detail of the signs of drowning, which are given with the author's usual accuracy and fidelity: for the presence of water and frothy mucus in the air-passages is not uniformly observed; and, when present, these marks are so appreciated as to lose much of their value as affirmative proofs; for the trifling quantity of water which is occasionally met with in the trachea, if in its natural fluid state, is supposed to enter after death; and, as to the frothy fluid, it may be a question whether it is not the produce of the bronchial membrane from previous disease, or secreted during the struggles of death from other causes. The proofs of death before submersion, which the body presents, are the presence of morbid lesions, traces of poisons, and the absence of those signs which usually denote drowning.

Notwithstanding all that has been written on drowning, and the many observations recorded and experiments instituted, with the view of explaining its pathological physiology, and assisting judicial inquiries, it is a subject still involved in considerable obscurity and difficulties, and requiring further investigation.

The remaining subjects of this chapter, *hanging*, *strangling*, and *smothering*, are treated in the same order observed while discussing that of drowning. We are presented, in the first place, with the characters which denote these modes of death respectively, or indicate a different mode of death from that assigned; and, secondly, the circumstances which are to be taken into account in determining the event to have been the result of homicide, suicide, or accident. Our limits will not permit us to follow our author in his details and discussion of these points; but they are full and satisfactory, and illustrated by several interesting and instructive cases.

The last chapter of this section of the work is devoted to the subject of *Wounds* and *Bruises*, injuries which constitute the most common causes of violent and accidental death, and furnish the most frequent occasion for the appearance of a medical witness in a court of justice. These may become the object of medico-legal investigation, either before the final event of the injury, or after death or recovery. In the former case, the witness will be required to describe the nature of the injury, and to pronounce on its tendency and probable consequences. In the latter case, he will have to state, where a fatal termination has taken place, the characters of the injuries found on the body, —whether they were inflicted during life,—whether they were the cause of death,—and, if so, whether they are to be charged to the account of suicide, homicide, or accident. In the event of recovery, he will be required to determine what degree of inconvenience or damage the patient has sustained.

The circumstances to be considered in forming an opinion of the consequences or danger, remote as well as immediate, of inflictions from external violence, are comprehensively detailed by the author, in some general preliminary observations. These refer principally to their characters, whether incised, lacerated, or punctured, &c.; their complication and extent; the parts of the body implicated; the modifications arising from peculiarities of constitution; the patient's state of health previous or subsequent to the receipt of the injury; and the mode of treatment pursued.

If the surgeon is required to be careful in his observation, and minute in his detail of circumstances, while the event is yet undecided, he is expected to be no less particular when the dead body is the subject of investigation. With reference to this part of the practitioner's duty, our author's observations relate chiefly to the mode of conducting the post-mortem examination, —the cautions to be observed in dissection,—the various particulars to be noted in his report,—and the physiological and pathological knowledge required of him in distinguishing morbid appearances from those of the healthy state, and estimating, in complicated cases, how far death is chargeable to the progress of disease or the effects of vulnerary lesions. Lastly, the circumstances which determine whether a wound was inflicted during life or after death are considered, with the discriminative characters of the discolorations which take place from animal decomposition after the extinction of life, and those which are caused by bruises on the living body.

The practical application of the foregoing and some additional observations, of importance in a judiciary point of view, will be found more particularly illustrated in the sequel, when the author is treating of wounds in the order of the parts of the body in which they are inflicted,—as the *head*, *neck*, *thorax*, and *abdomen*. *Gun-shot* wounds, as they possess some peculiarities, are treated of separately.

*Death from spontaneous personal agency, or suicide.*—In all cases of self-destruction, however evident it may be that the deceased deprived himself of existence, it is the custom to have the manner of death, and the fact of suicide, verified by professional testimony. In such cases the duty of the practitioner is comparatively easy; but there are other cases in which the investigation is attended with more or less difficulty. Instances occur in which a person takes away his own life, where his friends, naturally wishing to conceal the fact, will attempt to hide the real state of the case by ascribing the event to natural causes; and it may be the interest of survivors, as in cases of life-insurance, to establish that a person died from disease, when suicide is alleged or suspected: but the most important question, and the one which more frequently presents itself, is between *homicide* and *suicide*. The discussion of this subject is necessarily narrowed by the ample consideration bestowed, in the preceding section, on the different species of violent death, in their various bearings; but there are several circumstances to which our attention is here more particularly directed, as tending to elucidate the question between suicide and homicide. These are both of a moral (or circumstantial) and physical nature, and are introduced under the heads of *suicide*, by *poison*, *drowning*, *hanging*, and *wounds*.

Prolicide.—By this new term the author designates the destruction of the fœtus in utero, or *fœticide*, involving what is commonly called *criminal abortion;* and the destruction of the new-born infant, or *infanticide*.

*Abortion.*—The wilful and malicious procuring of abortion, or the administration of medicines, or any other means, *with that intent*, varies as to the degree of criminality attached to it by the law, and the punishment awarded, according to the stage of pregnancy at which the attempt is made: before the period of *quickening*, the criminal is considered guilty of felony, and liable to transportation for fourteen years; after quickening, the crime is punishable with death. Though this distinction is founded on the manifestly erroneous notion prevalent amongst the vulgar, and formerly believed by physiologists, that the embryo had no separate principle of animation till the period of quickening, yet, as it still maintains in law, considerable importance attaches to every variety of circumstances under which the procuring of abortion may call for investigation. On this account, the author enters into a detail of the growth of the embryo, and the various changes which it undergoes in its progress, from the earliest period after conception at which it is perceptible, to the completion of the seventh month, when it has acquired such a degree of development and perfection, as to render it capable, if separated from the mother, of being reared and attaining to old age.

The author lays it down as a general fact, the rare exceptions to which scarcely deserve consideration, that under the fifth month no fœtus can be born alive; from the fifth to the seventh month, it may come into the world alive, but cannot maintain existence. These are termed by the French *non viable*, by our author non-rearable, or *immature*,—in distinction to those born between the seventh and ninth month, which may be reared, and are merely *premature;* a child carried to the full period of utero-gestation only being considered *mature*. The term abortion is confined to the expulsion, which necessarily implies the destruction, of the *immature* fœtus; the question relating to the extinction of its life after the seventh month, being considered as properly belonging to the subject of infanticide.

The duty of a medical jurist in a case of abortion resolves itself, first, into the ascertaining of the reality of the event; and secondly, the determining whether it has been caused by natural means or improper interference. In conformity, therefore, with this view of the inquiry, the author goes into a detail of the phenomena of abortion; the causes existing, either in the mother or in the fœtus, which, without question of culpability, may induce it; and, lastly, the various means resorted to with a criminal intent. The different methods employed in order to excite the expulsion of the immature ovum, are considered under two heads,—those which act through the system of the mother, such as powerful medicines; and those which are at once applied to the uterus, such as violence and blows externally inflicted, and mechanical irritation directly applied to the uterus by means of instruments purposely constructed, or other expedients. The charge of resorting to these criminal means may affect the mother alone, or she may be an accomplice in the crime; but we must not forget that she may be altogether unconscious of the nefarious attempt practised upon her, and deceived to take medicines, in the persuasion that she labours under a natural disorder.

*Infanticide.*—The murder of a child newly born, or about to be born, has not only been visited with the severest punishment when proved, but until very lately, in this country, was punishable with death when only presumed. By a law passed in the reign of James I. it was enacted that *concealment of the birth* of a child, which, if born alive, would have been a bastard, was to be accounted satisfactory proof of murder against the mother ; and the evidence of one witness, at least, was required to establish the fact of such a child having been born dead. The extreme severity of this law, which defeated the purpose of its enactment, led to its repeal in the last reign, and the trials of women charged with the death of their offspring are now conducted on the same principles as other trials for murder; the jury, in cases of acquittal on the capital charge, having the power of finding, if made out in evidence, the fact of concealment of birth, which is punishable with imprisonment. To prove concealment of birth, it may be sufficient to ascertain that there has been a pregnancy or a delivery: to establish the guilt of child-murder, the body of the infant supposed to be murdered must be found. In either of these cases, the testimony of professional men may be required to establish points of the greatest importance to the parent, or others who may be implicated in the act of accusation.

In a case of alleged infanticide, it may be necessary to establish by professional men that a child has really been born alive, and that delivery has been suffered on the part of the mother. The consideration of the latter topic belongs to the subject of Pregnancy, where it is fully treated of by the author. With regard to the body of the child, the first thing to be inquired into is—whether the fœtus had been so long carried in the uterus as to attain sufficient powers to support life when separated from the mother: if it be established that the infant could not have maintained itself alive out of the maternal womb, or, in other words, that it had not reached the end of the seventh month of utero-gestation, the charge of murder must fall to the ground ; for, though a fœtus may come into the world alive before this period, experience has taught that it cannot continue to live. It is of the highest importance, therefore, to establish whether the fœtus has or has not passed the seventh month,

This leads the author into a detail of the peculiarities which distinguish the *immature* from the *rearable* and *mature* fœtus: such as the weight of the body, its measurement, colour, texture, disposition of the viscera, &c. If, again, the child is ascertained to be of the full term of utero-gestation, or so nearly approaching to it as to have been rearable, the questions for our solution are, whether it came into the world alive, and, if it did, what has been the manner of its death.

The appearances which the body presents when the child has been dead for some time previous to birth, are not to be overlooked; but what we must chiefly ground our opinion on, is the peculiarities in the circulatory and respiratory systems before and after birth. Hence the author presents us with an account of the mechanism of the circulation in the fœtus, and the anatomical peculiarities observable in the condition and appearance of the lungs and heart, compared with the changes which they undergo, and which are perceivable immediately after respiration has commenced. These afford the material evidence for concluding whether a child has been born alive or not, but each of the tests which they furnish has been the subject of objection and dispute among medical writers. The force of these objections the author thinks is greatly over-rated, and he is of opinion that much of the uncertainty alleged to belong to the subject of infanticide, has arisen from the acknowledged difficulty and labour of the necessary investigation, as well as the natural bias in the mind of the professional witness to favour the accused. On this account, he enters into a full examination of the various proofs afforded by the state of the lungs,—such as their specific gravity, or the *hydrostatic test;* their absolute weight, or the *static test;* and the proof proposed by Daniel, drawn from their *bulk.*

The *hydrostatic test* is founded on the difference of specific gravity, compared with that of water, between lungs that have respired and those that have not been distended with air. If, in the former case, they are thrown into water, they will float; and in the latter, they will sink: if we remove the lungs from a still-born fœtus, and place them in a vessel of water, they will sink to the bottom; but the lungs of a child that has made one inspiration will be buoyant. This fact, which was known as early as the time of Galen, was not applied to the elucidation of the subject of infanticide till the year 1660; from which time it was long considered a satisfactory test as to the birth of a living or dead child. Subsequent observation, however, having pointed out some fallacies to which the experiment was liable, and objections being raised to it, practitioners seem to have passed from the extreme of implicit reliance to that of unqualified distrust in its validity; and it has lately been declared in a court of justice, (and the authority alleged for the opinion is professional testimony,) to be not only absurd, but one that has been long exploded. Our author, however, while he admits that the test is too often applied in an absurd manner, contends that the real cause of the neglect of it is neither the absurdity of the thing itself, nor any authorised suppression of the practice, but rather a want of ability or of inclination to undertake the experiment as it should be performed. He then proceeds to a candid detail and examination of the principal objections that have been urged against drawing conclusions from the buoyancy of the lungs in water, and, after making what we conceive to be a fair estimate of their force, decides, with the best writers on juridical medicine, in favour of the validity of the test, provided the experiment is properly performed, and due regard is paid to all the circumstances which are said to disturb, in certain cases, the uniformity of the result.

The *static*, or *Ploucquet's*] *test*, founded on the difference in the absolute weight of the fœtal lungs, compared with that of lungs which have respired, was first applied to the detection of infanticide by Professor Ploucquet; and hence it generally bears his name. While the *specific gravity* of the lungs is diminished after respiration has commenced, their *absolute weight* must be increased from their receiving a larger supply of blood, in consequence of the change effected in the distribution of the circulation. Ploucquet, from the observations and experiments which he made, stated that the weight of the lungs of a full-grown fœtus which had never respired, was to that of its whole body as one to seventy; while in new-born infants, after respiration had been established, it was increased to two to seventy, or as one to thirty-five,—that is doubled. These experiments seem to have been too few to warrant the establishment of a rule from them; and of this Ploucquet was fully aware, for he expressly observes that his test cannot be received as an established proof, until a greater number of trials shall have been made,—their results accurately recorded,—and even a scale of proportions deduced between the absolute weight of the lungs and that of the bodies of children born at different periods of gestation. The subject has not been prosecuted, in this country at least, with the attention which it deserves; for, though no standard which can be implicitly relied upon has hitherto been established, yet, as the test rests on an incontrovertible physical law, it ought never to be neglected, because, admitting that several circumstances may alter the absolute weight of lungs that have not, and of those that have respired, a difference will still be found, which, when joined with the results obtained from the hydrostatic experiment, will furnish an additional link in the chain of professional evidence. The same may be said of

Daniel's proposed test, which is derived from the bulk of distended lungs, compared with that of lungs which have not respired.

Having largely discussed the proofs by which we are to verify the fact of the child's having been born alive or dead, the author next enumerates the various means by which it may have come by its death: whether chargeable to *omission*, or the neglect of those aids and precautions which the new mode of existence, as well as the feebleness of the infant, render essential; or to *commission*, or actual criminal interference; and concludes by calling our attention to the practical application of the foregoing facts and discussions.

The manner of conducting the inquiry in an alleged case of infanticide, is detailed with the precision and minuteness which the importance of the investigation imperiously demands. Before proceeding to the anatomical investigation of the body, the practitioner is directed to take an account of the adventitious circumstances and appearances about the child,—as the nature of the situation in which it was found; the state of the body as to filth or blood, &c. Its weight and measure is then to be ascertained, with a view to fix the probable period of utero-gestation. The presence or absence of putrefaction is also to be noted, or evidences which it may present of having died in utero, and having been afterwards detained there for some time. The surface of the body is then to be carefully examined, in order to detect any ecchymosis or wounds, particularly about the fontanelles and sutures of the head; and, as fatal luxations may exist, the state of the neck and cervical vertebræ is next to be inquired into, and the condition of the umbilical cord carefully observed.

In exploring the interior cavities of the body, much patience, deliberation, and order, is inculcated. By determining the import of one appearance, or the state of one organ, before he proceeds to examine another, the labour of the practitioner will be considerably abridged; and, what is of greater moment, the object of his research will be more effectually promoted.

After tracing the different steps of the examination of the body, in the order which ought to be followed, and laying down particular directions for conducting the experiments to which the lungs must in every case be submitted, the author concludes this part of the subject with briefly recapitulating the import of the appearances supposed to have been discovered.

"If the diaphragm be very convex towards the thorax; and the lungs of a dark-red colour, retracted from the anterior part of their cavities, not covering the pericardium, of a firm consistence, sink in water under every variety of trial, emit no sound when cut into, and effuse no blood; when, along with these circumstances, blood is discovered in the ductus arteriosus, and the foramen ovale of the heart is open, the conclusion must be that respiration has never been performed. On the other hand, if we find that the lungs fill their cavities, are of a pink or light-red colour, elastic to the touch, swim high in water, make a crepitating noise, and pour out florid blood on cutting into them, we have considerable proof that breathing has taken place: and if to these we should be able to add the corroborative result as to absolute weight, the mass of physiological evidence will be strong indeed. The mere fact of respiration not having been performed, is not, it seems, to be received as evidence that the child was not born alive. In this case, all we can do is to declare that we can throw no further light on the matter from professional research, and leave it to law and justice to deal with the case in their own way. We should nevertheless continue the dissection, as we may, perhaps, ascertain more positively from other appearances whether the child could have come into the world alive.

"If we discover that breathing has been performed, and consequently that the child has lived after birth, we are to pursue the investigation with a view to discover the cause of death; and, in its further progress, it will be conducted on the same principles as those that should guide us in examining the bodies of grown-up persons under suspicious circumstances. By keeping in mind the causes of violent death, we shall make a right use of the remaining parts of the body. (p. 378—379.)

Though the author is anxious to establish the incontestible character of the evidence which, in cases of infanticide, the skilful practitioner may draw from a careful examination of the body of the child, the reader will be gratified to find that his zeal is not restricted to the ungracious task of establishing the commission of a crime. In an article expressly dedicated to the subject, and in his account of the natural course of a concealed pregnancy, the author urges, with much force and feeling, various considerations in favour of the accused, which the practitioner should not overlook, and some of which are of essential importance as evidence; particularly the fact of the unexpected supervention of labour, and the sudden expulsion of the fœtus in situations where it may meet with instant death. A child coming into the world under these circumstances may receive such violence, by falling against hard bodies, &c. as shall immediately deprive it of life: the woman may not be able to give immediate alarm of the event; or, if the offspring is the consequence of illicit amours, believing that no assistance can avail in restoring it to animation, the idea of concealment for the sake of her reputation will more naturally arise; but, on the discovery of the corpse with marks of violence upon it, suspicions will as naturally attach to her, though guiltless of its blood.

The fact of delivery by surprise being unquestionable, we are bound to admit the possibility of the death of the child happening in this way, without interference or wilful neglect on the part of the mother, from violent contusions, suffocation, or rupture of the umbilical cord and fatal hemorrhage. We are also directed to allow their due weight to those appearances which are sometimes the consequences of a severe and protracted labour, and take care that we do not confound them with the effects of voluntary violence. In the prosecution of this topic, indeed, the author omits nothing which may operate in favour of the unhappy mother: even matters of a circumstantial nature, and, strictly speaking, extra-professional, which militate against her being guilty of such an unnatural crime, are pointed out for our consideration,—as her having made preparations for the care of her future offspring: thus the plea of delivery by surprise receives confirmation, if baby-linen be found in her possession.

We shall conclude the subject of infanticide with the introduction of a note from the author, on an erroneous notion, pretty prevalent among the vulgar, as to the lawfulness of getting rid of monsters as soon as they come to light:—

"It can hardly require discussion, but it may be adverted to in this incidental manner, that persons have conceived it warrantable to destroy infants born with such defects or monstrosities as to render their continued existence impossible, or their death desirable. Without arguing against the unwarrantable nature of the notion, I shall merely quote the observation of a learned judge at the York assizes in 1812, when two women were tried for drowning a child that was born with a deficiency in the cranium, in consequence of which it was likely that it could not survive beyond a few hours. There was no concealment on the part of the prisoners, one of whom was a midwife, and bore an excellent character for humanity. 'I think,' said his lordship, 'this prosecution may be of great use to the public, in removing an erroneous opinion, that the law allows the right of deliberately taking away the life of a human being *under any circumstances whatever.* It is, therefore, highly necessary that the contrary should be known.'

"The performance, however, of embryotomy, in order to save the life of the parent, (at least until the Cæsarean operation, or some other alternative, is established,) must not be considered as prohibited even by this statement from so high a quarter." (P. 384, *note.*)

The chapter on Infanticide is one of the most ably written in the book. The subject is presented in its most interesting aspect, and is treated with great power and impartiality. The rules laid down for conducting the investigation which justice demands will, if properly attended to, divest this sort of inquiry of many of its real and gratuitous difficulties, and lead to more satisfactory results than we are daily in the habit of witnessing.

With the subject of infanticide we shall also conclude our extended analysis of Dr. Smith's work. We wish we could follow our author through the other interesting topics which employ his pen, and which, though not equally important with the subjects already discussed, affect (many of them at least) interests of no small moment to the welfare and security of society, as well as the comfort, reputation, and liberty of individuals. The importance of questions involving the extinction of life under every variety of circumstance that may lead to judicial investigation, appeared to us to point them out as the subjects affording the best evidence of the qualifications of our author for the task in which he engaged. The specimens with which we have presented our readers have, we hope, borne us out in the favourable opinion we expressed at the commencement of the article, of the manner in which the work is executed. One reason of the incomplete view we have given of the subjects on which we have touched, consists in an excellence of the author to which we are glad to testify,—viz. the terse and compressed style in which he writes, and the total absence of expletives and repetitions, which render abridgment difficult, but, we need hardly add, constitute a valuable quality in a work of this kind. Those only who have attempted to peruse the ponderous and voluminous tomes on juridical medicine, which have issued from the continental press, can duly appreciate, and thankfully acknowledge, the labour and skill which has compressed into a comparatively small compass all that is valuable and satisfactorily established on the subject. As a book of reference in cases of difficulty and emergency, it will prove a valuable acquisition to the practitioner; and its merits as a composition entitle it to be registered as a creditable accession to British medical literature.

The remaining subjects, which are amply discussed by the author, but which we are obliged to pass over without any particular notice, are—Chap. II. Questions arising from injuries done to the person, not leading to the extinction of life, as *maiming* or *mutilating*, *surgical operations*, *corporeal punishment.*—Chap. III. Disqualifications for performing social or civil functions, as moral disqualifications, comprehending *mania*, *melancholy*, and *fatuity;* physical disqualifications for *general purposes*, for *military service*, and for *marriage*, &c.—Chap. IV. Miscellaneous questions, as *utero-gestation*, *sexual ambiguity*, *personal identity*, *survivorship and insurance of lives;* with some observations on *medical evidence*, the usages of courts of justice, and the manner in which the medical witness should comport himself there, and deliver his opinion.

Art. II.—*Observations illustrative of the Nature and Treatment of the prevailing Disorders of the Stomach and Liver.* By Thomas John Graham, m.d. Member of the Royal College of Surgeons in London.—8vo. pp. 224. London: Callow and Wilson, 1824.

Dr. Samuel Johnson observed, that a very entertaining work might be written upon the fortune of physicians, and perhaps that remark might be equally applicable to the fortune of diseases also. Whoever has looked upon the medical world with an attentive eye for the last twenty or thirty years, cannot but have observed the influence of fashion and imitation in the practice of the healing art; and never was this more fully illustrated than in the downfall of mercury in syphilis, and its establishment as an universal and general remedy for the cure of a disease, of which our ancestors did not dream, but which every man and woman, and many children indeed, are now constantly suspected of labouring under. Mercury may now be said to be a companion of the toilette; it is commonly used as a domestic medicine, prescribed by every *Lady Bountiful* in the country; and salivation and tenderness of the gums (terms formerly held disgraceful, and only to be whispered among the dissipated and debauched,) are now familiar in the mouths of the most delicate of our fashionable belles. An affection of the liver has become the prevailing malady: without affixing any precise idea to the term, it is generally considered as a sufficient explanation of all the symptoms, not of absolute illness, perhaps, but of discomfort and uneasiness, of which a patient may complain; and, to set all matters right, mercury is invariably resorted to, and sometimes pursued to a frightful and unwarrantable extent. It is true, that lately several signs have appeared of the commencing decline of these fashionable doctrines, and we are happy in being able to announce the work before us as one which, in our opinion, is likely to contribute much towards reducing the diseases of the liver within their proper boundaries, and turning the attention of patients, as well as physicians, to the consideration of those derangements of the stomach and intestines, which too often simulate the symptoms of liver disease, and are consequently confounded with it; a mistake likely to be attended with serious consequences whenever it occurs.

Dr. Graham, in his Preface, commences by asserting the superior importance of the stomach and intestines, contrasting their exquisite sensibility with the dull and comparatively little-sensible liver; and boldly asserts that nine-tenths of those complaints called *liver and bilious complaints*, are in reality affections of the stomach and bowels. It is, however, not against the use, but the *abuse* of mercury, which our author exclaims: he feels persuaded (we use his own words) that calomel has been the most fruitful of all sources of the astonishing frequency of stomach and (what are erroneously termed) liver complaints.

Our author, commencing his work with some pertinent remarks on fashion in medicine, proceeds to enumerate those symptoms usually ascribed to disordered liver: viz.—

"A sense of distention and oppression after eating, with flatulent, acid eructations; diarrhœa, or constipation, and uneasiness of the bowels; furred tongue; impaired appetite and strength; discoloured motions, they being either green, black, or much too light; nausea, head-ache, and bilious vomiting; palpitation of the heart; pain in the pit of the stomach and towards the right side; sallowness of complexion, and depression of the spirits:—and if the chief, or the whole, of these symptoms are present, especially if in a severe degree, it is usually considered sufficient to justify the opinion that a liver disease exists. But, according to my experience, a very large majority of those maladies are not liver complaints, but properly disorders of the stomach and intestinal canal; and this fact will form the subject of consideration in the first part of this Essay." (P. 5, 6.)

He readily admits that the liver is often secondarily affected; but then, so far from requiring mercurial treatment, it follows, as a clear consequence, that a plan of treatment adapted to the cure of the original affection will be most effectual.

We pass over several pages devoted to the consideration of the little natural sensibility of the liver, and illustrations of the great extent to which that viscus may be diseased, without either local pain being felt, or any sensible derangement of the health having been perceived; and these facts are contrasted with the acute sensibility of the intestinal tube, the strong sympathy subsisting between it and the remoter parts of the body, and the numerous and severe maladies which it simulates, and to which it gives rise.

The circumstances which have principally concurred to render hepatic disorders, and their remedy calomel, so common in England, are principally (says Dr. Graham) these—

"1st. A fulness and tenderness on pressure, and pain, being often present at the pit of the stomach, extending a little to the right side.

"2d. The alvine discharges being almost always discoloured in bowel complaints, and not unfrequently green or black, like pitch, from which they have been called *bilious;* and the power of small doses of mercury in correcting this appearance.

"3d. Organic disease being sometimes found in the liver after death, in cases of intestinal and other disorders, when no traces of such mischief are detected in any other viscus.

"4th. A great number of our countrymen annually return from the East and West Indies with biliary and intestinal disorders, arising from their residence within the tropics, where the liver is the organ the most obnoxious to disease, and where calomel is the sovereign remedy for all bodily ills: these, on their return to England, are ready to pronounce the maladies of their friends to be liver complaints, and cannot, of course, conceive any other medicine equal to calomel.

"5th. The sensible influence which the opinions and practice of professional men from India have had, and still continue to have, over medical practice at home." (P. 22—24.)

In reply to the first of these reasons, our author remarks that the situation of the stomach, duodenum, and colon, may well account for the tenderness so often felt upon pressure, and so uniformly referred to the liver; and for proofs of which he refers to the reports of cases, and examinations of bodies after death, particularly of two cases as reported by Mr. Howship. The occurrence of these pains a fortnight or three weeks only after the patient is first conscious of indigestion, constitutes another argument for supposing that the liver is not the seat of disease, since such a derangement would take a longer time to develop itself.

With reference to the discoloured state of the alvine discharges, which has been announced as a proof of diseased liver, our author observes, that, under disease, that power of the stomach, which Dr. Fordyce called its *governing power*, is lost or impaired; that an acid is generated, by which the bile is decomposed, and green stools are the consequence; in others, viscid and black evacuations take place, from the union of this acid with the soda of the bile. The immense quantity of offensive matter occasionally discharged under severe disorder of the stomach and intestines, he thinks, tends much to confirm this opinion; since the secerning vessels of the liver bear no proportion to those of the intestinal tube. The same remark also applies to black and bloody evacuations. This portion of the subject is pursued by our author through many pages, and he brings skilfully to his aid the evidence of M. Andral, and the testimony of Mr. Abernethy himself; whose work, ill understood, has been the fruitful source of mischief in the treatment of these diseases. In the course of these remarks, we find Dr. Graham paying a handsome compliment to the venerable Dr. Jackson, whom, with much justice, he represents as having anticipated, in his work on Fever, published in 1798, the correct pathology of fever, and having first suggested that rational and successful mode of treatment, by blood-letting, purgatives, free ventilation, &c. which is now so universally adopted.

Our author brings strong proofs of the correctness of his opinions from the writings of Drs. Pemberton, Blackall, and the cases published in the Medical Repository, as well as from the pathological labours of Andral, Broussais, and others, who have not been able to detect disease of the liver in any thing like the proportion with the frequency of intestinal lesions.

What degree of credit to give to our author's fourth reason for the fashionable doctrine of liver disease, we hardly can determine; but we should think that the influence of our valetudinarians from the Indies not quite so great as he imagines. Nevertheless, his observations on the different powers of medicine in different climates is perfectly accordant with our own experience; and we can safely say that, if the Italians are surprised at the doses of some medicines as given in England, our astonishment is not less at the vigorous manner in which they employ the tartar emetic, and some other potent remedies.

We are well inclined to believe that the practice of many men of talent, who have been accustomed to the acute hepatitis of India, and the consequent free use of mercury, has had a considerable influence in this country, and a very pernicious one: and the difference observable in the effects of large doses of that medicine in different quarters of the globe, is not among the least curious pathological facts; and the disregard of this plain truth has been the groundwork of much mal-practice, in this country especially.

How much appearances may deceive, and how necessary it is to be guarded in prognosis, the following case will show, and which we shall transcribe, although it has appeared, at no distant period, in the pages of our respected contemporary.

"Richard Sutton, ætatis twenty-five, servant in husbandry, was admitted into the Canterbury hospital, April 18th. This poor fellow was in a very debilitated state, and could not give any account of himself. From a person, however, who accompanied him, I learned that his symptoms were 'sickness, inability to retain any thing on the stomach, very obstinate constipation,' and that he had some time before laboured under fever and inflammation.—Habeat quam primum hydrarg. subm. gr. x. Extr. hyoscyami gr. v. Inj. enema purgans. Pil. hydrarg. gr. v. P. ipecac. comp. gr. x. horâ somni. On visiting him next morning, I had leisure to make a closer examination. Skin of a yellowish green hue, as were the conjunctivæ, (as described by Dr. Baillie, in green jaundice.) Great prostration of strength, and flatness of the abdomen. Pulse scarcely perceptible at the wrist. No fulness of either hypochondrium. On applying pretty severe pressure to the right lobe of the liver, he appeared to wince. Urine natural in quantity, but rather highly coloured. The calomel has procured several dark, offensive stools. Sickness only after eating.—Retained the Dover's powder, which, with the blue-pill, is to be continued every night. Cathartic mixture every morning, and the effervescing mixture occasionally.

"This plan was persevered in until the 25th, during which time I had several opportunities of showing this case to my professional friends, who agreed with me in thinking (though the case was obscure) that the *seat of the disease was the liver.*

"25th.—Omitt. Pulv. ipecac. co. Cont. Pil. hydrarg. Illin. semidrama Ung. hydrarg. Fort. sup. reg. Hypochon dextr. quâque nocte.

"The greatest attention was paid to the different symptoms. The bowels became more regular in their action, and the dejections more natural; the sickness, too, was less distressing. Nourishing food, with wine, was given; as well as bark, aromatic confection, &c. &c. The treatment, however, was of no avail: he died on the 20th May.

"*Dissection*.—I examined the body twenty-four hours after death, when I found *the liver perfectly natural in size and structure; the gall-bladder about one-third full of healthy bile*; the stomach smaller, and more flabby than common; no disease of the cardia or pylorus; pancreas, spleen, kidneys, and urinary bladder, natural. The intestines had a contracted appearance, and their villous coat, as did that of the stomach, readily yielded to the application, though slight, of the finger-nail. The lungs were studded with tubercles in different stages, and very firmly attached to the pleura costalis on both sides, requiring very great force to separate the adhesions. The pericardium contained about an ounce of fluid, and was here and there spotted with coagulable lymph on the internal membrane. I thought the heart was smaller and softer than natural, but could not discover any disease in the mitral, semilunar, or tricuspid valves; neither was there any communication between the ventricles; the foramen ovale was closed. On removing the skull-cap, I was astonished to find the vessels, even the most minute, gorged with blood. The ventricles contained more fluid than usual, and there was evidently a softening of the centrical and medullary substances." (P. 123—127.)

After making some further observations upon other symptoms usually ascribed to affections of the liver, we come to our author's own more peculiar views of the subject. There are (he says) three different kinds of disorder of the digestive organs, each having its seat principally, if not exclusively, in a particular organ, and requiring a somewhat different treatment; though one species seldom occurs, and exists for any time, without in some degree occasioning the others. These three complaints are—those of which the stomach is the seat; those in which the intestinal canal is principally concerned; and, thirdly, those where a faulty or deficient biliary secretion is the principal or only complaint.

The first class of complaints is denoted by furred tongue, want of appetite, oppression at the pit of the stomach after meals, &c. The mouth is parched and dry in the morning; there is thirst, and the breath is offensive. The bowels sometimes are regular, often costive; there is tenderness on pressure at the pit of the stomach, and sometimes on the left side; morbid acidity in the stomach is also common; the urine is turbid, and deposits a yellowish, or yellowish-red, sediment. The complexion is pale, but rarely sallow. Such are the principal symptoms.

When the intestinal tube is the affected part, there is no fur on the tongue; the appetite is often more voracious than usual; there is no thirst, no fœtor of the breath, nor pain at the stomach:

but the bowels are irregular in their action, the stools are offensive, and diarrhœa is often present. When there is pain, it is felt on the right side; sometimes piles are troublesome; at others there is tenesmus. The countenance is often yellow, and the conjunctivæ also. In both these forms of disease, the pulse is unaffected in general.

In the third instance, where the biliary organs are chiefly affected, there is constipation; unhealthy evacuations, white or black; pain on the right side; a yellow, thick fur on the tongue, and high-coloured pink urine. The pulse is but little altered. Digestion in the stomach seems to be tolerably perfect; but indigestion begins below that organ. The yellowness of the eyes and countenance may be more permanent, but not more general than in the disorders above mentioned.

Of these three forms of disease, the second appears to our author to be the most common; those of the biliary function the least so.

We have now gone rapidly through the first part of Dr. Graham's publication, and come next to the consideration of the mode of treating these forms of disease, which it will be seen constitutes, in fact, the principal novelty and merit of the work; and here we are glad to perceive that, in regulating the exhibition of mercury, our author does not run into the common error of those who adopt peculiar views, of discarding entirely the medicine which has been over-rated or misapplied, but merely urges the exclusion of one particular form of it, under certain circumstances, and the more mild and sparing use of it in others. We shall not transcribe our author's accumulated proofs of the irritating qualities of calomel, when too frequently and too largely exhibited. We are prepared, indeed, to go further in the condemnation of this practice than the author himself. How often have we not known the quantity and colour of the evacuations produced by a large dose of calomel, insisted upon as the best reason for repeating it? But it is most certain that the extent and quality of the discharge is commonly the product of the medicine itself, and has no reference whatever to any condition of disease: nay, the oftener it is repeated, the stronger will be the apparent necessity for its repetition. But the real question is, in what particular affection is it necessary to prescribe mercury at all? Our author is of opinion, that minute doses of tartrite of antimony and rhubarb combined will, in very many instances, be equally efficacious in remedying the disease, and will have no detrimental effect upon the constitution; and nothing can be more true than Mr. Abernethy's axiom, now totally disregarded, " that, *if an unhealthy condition of the bile is induced by the stomach, no blue-pill will avail.*" Still our author candidly admits that sometimes calomel may be eligibly administered; but he cautions us against an over-dose, and he brings a host of medical testimony in favour of this view of the subject, but whose opinions seem to have been wholly overlooked in the universal rage for mercury that has lately prevailed. We fancy that many of our readers will scarcely persuade themselves that neither Mr. Abernethy nor Dr. Farre are advocates for continued or large doses of mercury; and that men of great reputation have ascribed, and apparently with reason, many of the protracted diseases of children to the very remedy intended for their removal. Nay, it may be questioned whether struma itself may not be called into action frequently by such violent means.

This subject is pursued through many pages; but we now revert to those remedial agents which Dr. Graham substitutes for mercury. He considers that, in certain morbid affections of the stomach, the nitric acid is an invaluable remedy; and in others he recommends Brandish's caustic alkali, originally used in scrofulous complaints. The nitric acid he thinks chiefly beneficial in those cases where the stomach and duodenum are principally affected, and especially where mercury has previously been largely exhibited. It may be taken to the amount of six drops three times a-day, and gradually increased to eight or ten drops.

The caustic alkali of Brandish is also of most service in stomach disease; but, instead of being applicable where *heat* is a troublesome feeling, it is where coldness of the feet, languor, chilliness of the surface, and morbid acidity prevail, that its good effects are most sensible. It should be given in doses of a tea-spoonful morning and evening, and gradually increased to two tea-spoonsful. Its taste is best concealed by beer or milk, and it should be greatly diluted. Of course, the use of bitters and tonics is not precluded.

With regard to aperient medicines, our author, of course, condemns strong purging, and prescribes some slight aperient pills, composed of colocynth, rhubarb, and ipecacuanha, or some purgatives of the same class, to be used as required.

When the bowels are the chief seat of disease, tonics are of inferior utility; the great indication being to carry down the residue of the food, and to excite healthy secretions from the internal surface of the bowels, for which purpose several forms of saline aperients are given. At the same time, our author admits that, in this modification of disease, the blue-pill has been very useful; but it should be prescribed in small doses. The compound calomel pill he also declares to be an excellent alterative in these complaints. If considerable irritation and uneasiness exist in the bowels, and they are not relieved by the above means, he advises the exhibition of the ol. terebinthinæ, according to the following formula:—

℞. Ol. Terebinthinæ ℨiv.

Vitell. Ovis q. s.

Sacchari albi ʒvj.

Ol. Menthæ gtt. iij.

Aquæ puræ, ℥v. M. cujus cochl. amplum bis vel ter in die sumatur.

The Cheltenham or Leamington waters may also be taken with great effect in the second and third class of disease of the assimilative organs. But it is in the third form of disease, or that wherein the biliary organs are chiefly affected, that mercurial preparations are most useful; but here even it should be administered with caution, and salivation carefully avoided, and aperients, or rather purgatives, may be more freely given. An occasional emetic may be useful; and the vapour-bath is decidedly so.

We must draw this article speedily (we had almost said, unwillingly) to a conclusion; but our limits will not admit of our saying much more upon the subject.

Our author next adverts to the virtues of rhubarb, especially in the bowel complaints of children. He then gives some excellent dietetic rules. He advocates the use of the warm bath, or sponging the surface of the body daily with tepid water; and urges the necessity of attention to local pain, when occurring as a symptom of any form of these derangements.

In conclusion, we sincerely recommend the perusal of Dr. Graham's work to our professional brethren. We have long been convinced that such a work was imperatively called for: it will unquestionably draw the attention of the young practitioner to the consideration of the merits of a system, which has now become universal in extent, and formidable from the energy with which it is universally resorted to,—the system of pouring in mercury upon the slightest, and often indeed without the slightest, pretence whatever.

### DIVISION II.

### FOREIGN.

ART. IV.—*Du Froid, et de son Application dans les Maladies; Considerations Physiologiques et Therapeutiques, Observations, et Corollaires.* Par S. TANCHOU, Docteur en Medecine de la Faculté de Paris, Membre de la Legion d'Honneur, &c. &c.

*On Cold, and its Application in Diseases; Physiological and Therapeutical Considerations, Cases, and Corollaries.* By S. TANCHOU, Doctor of Medicine of the Faculty of Paris, Member of the Legion of Honour, &c. &c.—8vo. pp. 131. Paris: Crevot, 1824.

THE application of cold as a remedial agent is, we believe, better understood, and more frequently employed, in this country, than with our continental neighbours. Cold water has been lauded and recommended by many eminent men in this country, and has been employed, in fever especially, to an extent which the author before us seems not to be aware of. He, indeed, mentions the work of CURRIE; but evidently does not know how extensively the plan of affusion recommended by him has been acted upon in this country. Nevertheless, we are not disposed to quarrel with M. Tanchou for his sins of omission: he has produced a little work, the general tenor of which is praiseworthy; many of the remarks with which it is interspersed are fraught with good sense; and his treatise may be perused with pleasure and profit, even by the experienced practitioner.

A short introduction developes the plan of the work. Our author is not one of those old-fashioned prescribers who are fond of a farrago of remedies; he boldly advocates the use of the most simple means of cure, and justly observes that, *a priori*, he should have conceived that diseases from over-excitement were the most common, since nature has been so prodigal of the means of repressing them; and, among these means, he considers cold as being the first.

The plan of his treatise is the following: first, general considerations, containing some observations on life and the living principle; secondly, an account of the effects of the application of cold in various diseases, with illustrative cases; and, lastly, a set of twenty-four Corollaries, or short aphorisms, arising out of the observations and discussions in the former portions of the work.

We shall not detain our readers with any extracts from the general considerations of our author. He evinces an intimate acquaintance with the principal medical theories which have succeeded each other from age to age, and which have always taken their tint from the philosophy of the day. We may also pass by the consideration of what cold is, and proceed to inquire into its effects when applied to the body in health: these are so well known, as not to need enumeration. The reaction consequent upon the application of cold, says our author, depends upon the degree of vital energy of the person: to the aged, to weakly persons, and children, cold baths are constantly hurtful; and therefore ROUSSEAU committed a great fault in recommending all children, without exception, to be plunged in cold water, and bathed in it daily. In disease, the two effects of cold may be advantageously employed; but every thing must depend upon the mode in which it is used.

Commencing with inflammation, our author observes, there are but three ways of combating that disease:—1st. By sanguineous depletion; 2dly, by suffering them to expend themselves *(de les laisser en quelque sorte s'user)* upon the part which is the seat of inflammation; or, 3dly, to displace it by revulsives. The first plan succeeds only completely when used at the commencement of maladies; the second is not without danger; but the third is applicable to all morbid alterations, only it must be graduated according to their intensity; and it is necessary to know those cases in which it would be prudent to attempt it.

All the means of cure that have been devised since the days of HIPPOCRATES to the present time have been, in fact, revulsives: such even now are the contra-stimulants of the Italians, *our little* blisters, our sinapisms, and even our insipid and nauseous tisans.

In nervous diseases, continues M. Tanchou, there is only one curative indication,—that is, to extinguish the exalted sensibility which is the cause, and forms the prominent character, of the disease; and cold stands foremost among the remedies for this purpose, acting, in the first place, by diminishing the general sensibility, and sometimes secondarily by forming, in consequence of the reaction, local congestion and inflammation. Such also, in this latter complaint, is the *modus operandi* of cold.

In slight maladies, especially where the nervous sensibility predominates, cold, by diminishing that sensibility, restores the balance of health. One example is given by our author, the case of Madame D., a highly nervous lady, who, without any marked symptom or real disease, suffered considerably from trifling causes. This lady had been accustomed to use baths heated to ninety-eight or one hundred degrees: her cure was effected by gradually reducing this temperature, until the patient was enabled to plunge herself into the coldest water, even in the winter; and it is remarkable, says M. Tanchou, that, although frequently suffering before this from colds, during the time that she employed the cold-bathing she escaped them entirely.

Several examples are given by our author of the good effects of the application of cold in some of the most formidable diseases to which humanity is subject. In the plague at Moscow, in 1771, Samoilowitz employed it with great success. Cirillo, of Naples, used no other remedy in fever than cold water as a beverage; and, finally, Currie and Giannini recommend the affusion of cold water in the same diseases. Paulini, Skragger, &c. have been equally successful in the treatment of intermittents. In the typhous fever, so frequently epidemic in armies, the good effects of cold water have often been remarked. We can ourselves vouch for the truth of this remark, and are in possession of a host of evidence upon this part of our author's subject. He illustrates his remark by several apposite cases; and then proceeds to discuss the mode in which the remedy may be supposed to act. Here we find our author engaged in controverting the doctrines of M. Broussais; and we shall take leave to pass over this (to us) uninteresting discussion, and proceed to matters more practical; first premising that our author's theory of the effects of cold is, simply, that it always acts by depriving parts of their caloric. This privation, as far as concerns the nerves, merely lowers their sensibility; but upon the capillary vessels it has also the effect of constriction, and consequently restraining the circulation, and, by this double effect, opposing inflammation.

We have now arrived at that part of M. Tanchou's work which relates to the application of cold to particular diseases. In every case it is adviseable to abstract blood previously to the use of this remedy, especially if the inflammatory symptoms are violent. In inflammation of the brain or its meninges, its application is not unattended with danger, unless it is continued until the disease is totally overcome; since its previous removal would be attended by a most severe reaction. It is needless to remark, that most extensive sanguineous depletion should be practised in the first place.

A strong case is detailed by our author, illustrating the danger of removing the ice from the head of a phrenitic patient, who, though much relieved and rendered tranquil by its application, was still but imperfectly restored. In two hours after its removal, the symptoms returned with increased violence. It was impossible to bleed the patient; four men could scarcely retain him in bed; and, in less than three hours from the renewal of the attack, he expired.

At page 49, we have some very wholesome rules relative to the use of cold applications to children. Our author observes, that strong children only are benefited by cold, and these are less affected by cold and catarrhs than others. In the use of this remedy in the cerebral inflammation of infants, it must not be forgotten that the bones of the cranium are very thin, not completely formed, and that there is little or no hair. In the case of convulsions in children, it is necessary to understand clearly the cause of the disease, before we have recourse to this remedy; since, in the case of colic or bowel complaint, its employment must be pernicious: and in no case should it be applied without premising an evacuation of blood. We extract the following case, as a specimen of our author's mode of management.

Miss Des——, aged five years, had been indisposed for five or six days. She was naturally of a florid complexion, very lively, forward of her age, and any resistance to her inclinations caused her to become convulsed. One day, after having been much irritated by contradiction, she was seized with pain in the head, the cheeks became alternately flushed and pale, and convulsive motions of the limbs were perceived. A medical pupil, who visited at the house, recommended warm bathing to the feet and injections; but nothing was done. The next day, all the symptoms were aggravated; fever came on, followed by delirium, and, in fact, all the symptoms of arachnitis. Leeches, sinapisms, and poultices, were employed, but without success; and, when M. Tanchou was called, he found the child lying on its back, the head a little thrown back; the eyes fixed, sensible to the light, and half shut; the pulse was quick, sharp, and contracted; convulsive movements of the limbs, face, and about the lips, were perceptible. It was proposed to renew the application of sinapisms and blisters; but our author wished previously to try the effect of ice: for this purpose, the patient was placed in a chair, the shoulders covered with napkins and cerecloth, to preserve the parts from moisture, and then the head was uncovered. He then began to pour upon it water of the temperature of the room; then he applied water just drawn from the well, and after, that into which some ice had been thrown. By degrees the patient's head, which hung upon the shoulders, resumed its proper position; the eyes opened; and, after about an hour of this application, the child knew and called her mother. The same application was continued for a few minutes; and afterwards the patient was placed in bed, and then the head was covered with ice: a person was placed on each side, to prevent the cap of ice from falling off, and to renew it from time to time, without removing it upon any pretext whatever. As soon as this was done, the blisters and sinapisms, previously recommended, were applied; and four days after the patient was convalescent. It ought to be observed, that the use of ice was continued for twelve hours; that cold applications were not entirely left off for two days, and that the temperature was during that time gradually increased.

In *gastritis*, our author, instead of employing tisans, orders the patient to hold a piece continually in the mouth; but this does not, of course, preclude the employment of other antiphlogistic means. In *nervous pains of the stomach*, also, we find cold insisted upon as the only remedy; but here we are cautioned against large draughts of liquid, lest vomiting should be provoked: and, as this part of our author's doctrine may appear as questionable to our readers as it does to ourselves, we shall add an account of his method of using his favourite remedy in a case of this description, and which, we think, will prove how little cold had to do in the cure of this particular malady. It will also show that our worthy author is not more free from the suspicion of riding his hobby too hard, than others have been before him.

Madame H—, a very nervous lady, just recovering from rather a severe affection of the chest, was seized with spasm of the stomach and vomiting in the middle of the night. She had complained, in her walk the previous evening, of cold in the feet, but had dined well; in fact, had eaten more than usual. Our author found his patient labouring under extreme agitation, pallid, with perpetual vomiting and hiccough; the tongue moist and foul; the matters ejected were mixed with bile, but had no trace of food; the abdomen was soft, and free from pain; the pulse small, calm, and undisturbed; the respiration was affected; the hands and feet cold, and a shivering was occasionally felt. Our author administered antispasmodics, and opiate frictions upon the pit of the stomach, (ether had already been taken;) the feet were warmed, and iced drinks were ordered. The following day, the symptoms were a little alleviated, and the pulse quickened. In the evening, acetate of morphine was given, and the patient passed a tranquil night. The next day, however, at the same hour, the symptoms recurred; and M. Tanchou thought the best way of subduing the disease would be to provoke fever, as he terms it: in fact, to produce external irritation by the application of a large sinapism to the region of the stomach. At the end of two hours, a *smart fever* took place, and all the symptoms disappeared. After an interval of two days, their return was threatened; but an irritating anti-emetic brought back the feverishness, the nervous train of symptoms were overcome, and the patient got well.

We are at a loss to conceive how any man of understanding could for a moment imagine that this malady was in any way influenced by cold water. Ether, opium, antispasmodics, mustard poultices, had all been called into action; and yet our author brings forward this case as a proof of the efficacy of iced drink.

We pass by a few pages, in which our author details at great length his own case, which appears to us to have been merely a partial paralysis of the fingers from cold, superinduced probably by some gastric irritation, but which he prefaces by some remarks upon what he calls an unknown disease.

He next relates the case of a lady, who appears to have suffered, after her confinement, from severe dyspeptic symptoms, and who seems to have owed her cure to a very light and spare diet, but which was always taken cold; and, therefore, our zealous author has no scruple in ascribing all the happy results to the cold itself.

In *peritonitis* of every description, including, according to M. Tanchou, puerperal fever in all cases and under all circumstances, cold is the remedy *par excellence.* For our own parts, although inflammatory action appears to be the essence of puerperal fever, yet there is at times something more than mere inflammation connected with that formidable disease; and therefore we cannot quite agree with our author in this compendious method of deciding upon an universal mode of cure. It is fair, however, to observe, that our author freely advocates the use of the lancet or leeches, though he very properly prefers the former: but he goes on to say, "If by these means I cannot make myself master of the inflammation,—if the pulse remains small and frequent, the belly painful and tumid, respiration affected, &c.—I employ ice: this method has always succeeded with me, and I have *never lost any patients* labouring under this disease." (P. 72.) A fortunate case is given in illustration, and in which the ice was applied, with the same precaution as to gradually lowering the temperature as in the case before detailed.

In *affections of the chest*, our author never employs or recommends the application of cold; but he mentions several instances of persons who used no other method of cure in cases of cold or catarrh. It must, however, be admitted that, where there is a tendency to hemoptoe or phthisis, such a plan must be precarious,—we might say, indeed, pernicious.

In *gout* and *rheumatism of the joints*, our author does not appear to be apprehensive of the consequences of the employment of his favourite remedy. All he premises is bleeding the part with leeches.

In *aneurisms of the heart and great vessels*, M. Tanchou observes that he has seen, in an aneurism of the aorta, the bursting of the vessel suspended for many weeks by cold applications; and, in incipient aneurisms of small vessels, the employment of ice has reduced them entirely.

A page or two is next devoted to the use of ice in *inflammation of the testicle*, which we should not have alluded to, but for the purpose of remarking that our author, like a true Frenchman, attributes the use of cubebs in gonorrhœa to M. Delpech, and balsam of copaiba to M. Ribes. Indeed, throughout the whole book, he appears to be totally ignorant of every English work applicable to his subject; or, at least, he does not mention them.

The next disease mentioned by our author requires a little consideration. It relates to those pains occasionally experienced by females prior to the menstrual discharge, and for the cure of which our author recommends cold glysters to be administered, at the time when the pain is most violent; and he enters into a long train of reasoning to prove the value of this remedy. For our parts, we shall be satisfied with the report of one or two cases in which it has been employed with success, premising that we have found camphor in substance almost a specific in this description of complaint. The case our author quotes was certainly highly successful, since the patient, who had been constantly troubled with these pains for three or four days prior to the menstrual period, was cured by one cold injection, and never suffered uneasiness afterwards. M. Tanchou adds, that he has subsequently used these means, and generally been equally fortunate. But we cannot but be struck with the concluding sentence of this page, and which we think it our duty to translate:—"I hesitated a long time to employ this agent: it appeared so opposite to those usually recommended by all authors and physicians, that it required mature reflection, and a desperate case only could decide me to adopt it. In spite of this, considering the danger that might ensue, I cannot even now venture to recommend it." (P. 96.) In this palinodia we beg heartily to join.

We have now arrived at the second part of M. Tanchou's work, which treats of the application of cold in external complaints attended with wounds. We shall make short work with this portion of our author's labours, since we find little novelty in his remarks, and are quite sure that, surgically, the use of cold water as a remedy is well understood and duly appreciated in this country. We must, however, protest against its general use in erysipelas, particularly of aged persons, or when situated on the head or face. It may be proper enough, as M. PINEL says, to do nothing in the way of external application; but, whatever theorists may say of metastasis, no practical man, we should conceive, would in such cases venture to apply cold water or ice.

One disease only remains to be mentioned, and that is *cancer*. We need scarcely say that nothing satisfactory is advanced by M. Tanchou upon this subject.

We shall conclude this article by transcribing a few of our author's corollaries: they are too numerous to give at full length; but the first eight are merely plain affirmations of the common effects of cold, and the usual phenomena of a shivering fit. The 9th Aphorism asserts that, in nervous diseases, cold, by taking away caloric, destroys the sensibility of parts; and that, in diseases of weakness, cold applied externally, at intervals, may be useful, by the successive reaction it produces; only it must be graduated to the vital power of the patient.

The 15th Corollary asserts that cold is salutary in asphyxia from carbonic vapours, but hurtful in those of submersion. The 16th recommends the *progressive* application of cold in acute inflammations, and the same progressive decrease at the termination of the disease. The 22d recommends that, wherever there is plethora or fever, general or local blood-letting should precede this remedy; but that in nervous affections this is not necessary, and may even be hurtful. The last Aphorism is this:—"The use of cold should never be given up until you have become master of the disease; the intensity must be measured by that of the complaint; and the lowest temperature must be reserved for the most severe cases."

---

ART. IV.—*Competitio ad Aggregationem jussu Regis optimi et ex Mandato summi Regiæ universitatis Magistri, instituta anno* 1823. *An in Curandi Oculi Suffusione (vulgo Cataracte), Lentis Crystallinæ Extractio hujus Depressione Præstantior? Theses quas, Deo favente, in saluberrimâ Facultate medicâ Parisiensi, præsentibus competitionis judicibus, publicis competitorum disputationibus subjiciet et dilucidare conabitur die 26 Februarii, anni* 1824. Auctor J. CLOQUET.—4to. pp. 9. Parisiis, 1824.

*A Comparison of the Operations for Cataract by Couching and by Extraction; being the Subject of a Thesis, to be defended and illustrated in a public Competition.* By M. J. CLOQUET.

THE author does not enter into the question of what cases require operation; but gives a comparative view, resulting from his experience and observation, of the dangers of two of the operations for cataract, viz. couching and extraction. The operation of destroying the cataract by absorption is not, in the whole paper, alluded to. He divides the contents under three heads:—1st. Of the accidents common to both operations; 2d, of these cases in which one operation is preferable to the other; and 3d, of the comparative success of both operations.

I.

1. *Couching is more easily performed:* although the favourers of the operation by extraction say otherwise. In both, the time for deciding to operate is the same. Of the evils which are alike common to the two operations, he gives formally the following list, with remarks:

1st. *Pain.*—Depression of the cataract with the needle seems the more painful operation, from the suffering of the generality of patients.

2d. *Injury of the Iris.*—It is liable to be hurt in both ways of operating. If in extraction, it is while puncturing and cutting the cornea; while opening the capsule of the lens; while the lens is protruding through the pupil. If in couching, it may be punctured by the point of the needle.

3d. *Hæmorrhage* may occur in either case, from wounding the iris, or in piercing the choroid coat.

4th. *Vomiting* is of rare occurrence in either operation; but is most dangerous after extraction.

5th. *Inflammation* produces most bad effects after extraction, though the dangers after both are very great.

6th. *The growth of an opaque membrane in the eye* succeeds extraction the most frequently.

7th. *The closing of the pupil* is common to both.

2. *Concerning the dangers which are peculiar to Couching.*

1st. *A scar upon the sclerotic,* is not of great importance.

2d. *The danger of wounding the ciliary ligament and the ciliary nerves;* either of these accidents may be easily avoided.

3d. *The rising of the lens* after having been depressed, is seldom met with: a milky effusion within the capsule, or a secondary cataract of the capsule, may lead to that supposition.

4th. *Pain, irritation,* and *inflammation,* may affect the membranes, especially the retina, after this operation.

5th. He informs us that the *protrusion of the cataract,* during the operation, through the pupil *into the anterior chamber,* is a slight accident. It is bathed in the aqueous humour, and becomes absorbed.

6th. A considerable time is required to complete a cure after this operation.

3. *Concerning the dangers which are peculiar to Extraction.*

1st. *Too small, and too large, incisions of the cornea:* the former prevents the cataracts escaping; the latter endangers a loss of the humours, and gangrene.

2d. *A white cicatrix,* not always destructive of sight, may remain upon the cornea.

3d. *The pressure of the ball,* in squeezing out the lens, brings on inflammation.

4th. *A protrusion and a loss of some of the humours* may happen to the most expert surgeon; and blindness is then almost inevitable.

5th. *The falling forwards of the iris,* is a serious occurrence, and is productive of many dangerous evils.

6th. *Staphyloma* of the vitreous humour and hyaloid membrane occurs after extraction: the consequences are, sometimes, fistula of the cornea and wasting of the ball of the eye.

7th. *Admission of air* into the chambers: this, he says, is one source of irritation.

8th. *The lower eyelid being admitted into the incision,* is difficult to obviate, and is a source of irritation to the eye, and of loss of its humours.

9th. *The irritation of the tears* causes inflammation.

10th. After extraction has been attempted, *no other operation can be again tried:* this is not the case with couching.

II.

*When one operation ought to be done in preference to the other.*

1st. If the patient be very irritable—*couching* is best.

2d. If the ball of the eye project much, or if the contrary be the case—*couching.*

3d. If the pupil be very small, and does not yield to belladonna—*couching*.

4th. If there be leucoma, nebula, pterygion; or if the veins of the eye be varicose, and the lids prone to inflame—*couching* rather than extraction.

5th. If it be the floating cataract—*couching*.

6th. If it be the very soft, cheesy cataract—*couching*.

7th. If the capsule of the lens adheres to the iris—*couching*.

8th. If there be dropsy of the eye, neither ought to be done: if any—*couching*.

9th. If there be a small-sized cornea—*couching*.

10th. If the catatact have increased in size—*couching*: if it be small—*extraction*.

11th. If we are dubious as to the nature of the cataract—*couching*.

12th. If there be staphyloma of the sclerotic, neither should be done: if any, *extraction*.

III.

In comparing the success of the two operations, couching appears most favourable. In 246 operations, 166 were performed by couching: 45 were unsuccessful; 121 were nearly successful. Eighty had the cataract extracted: 38 were left blind; 42 nearly cured.

He concludes, that neither operation is exclusively to be preferred; but the easier and more generally successful one ought to be practised most frequently. He prefers, by the drift of his paper, the operation of couching; although in his Thesis, by the mistake of the printer, the word *extraction* is made use of instead of *depression*.

